# Carers’ Motivations for, and Experiences of, Participating in Suicide Research

**DOI:** 10.3390/ijerph17051733

**Published:** 2020-03-06

**Authors:** Myfanwy Maple, Sarah Wayland, Rebecca Sanford, Ailbhe Spillane, Sarah Coker

**Affiliations:** 1 School of Health, Faculty of Medicine and Health, University of New England, Armidale, NSW 2351, Australia; swaylan2@une.edu.au; 2 Sydney School of Health Sciences, Faculty of Medicine and Health, University of Sydney, Sydney, NSW 2006, Australia; 3 School of Social Work and Human Service, Thompson Rivers University, Kamloops, BC V2C 0C8, Canada; rsanford@tru.ca; 4 National Suicide Research Foundation, Western Gateway Building, Western Road, Cork T12 XF62, Ireland; ailbhe.spillane@ucc.ie; 5 School of Public Health, University College Cork, Cork T12 XF62, Ireland; 6 Formerly SANE Australia, South Melbourne, VIC 3205, Australia; skcoker76@gmail.com

**Keywords:** suicide attempt, carers, lived experience, research participation, ethical research

## Abstract

(1) Background: First-hand accounts of lived experience of suicide remain rare in the research literature. Increasing interest in the lived experience of suicide is resulting in more opportunities for people to participate in research based on their personal experience. How individuals choose to participate in research, and their experience of doing so, are important considerations in the ethical conduct of research. (2) Methods: To understand the experience of providing care for someone who has previously attempted suicide, a cross-sectional online community survey was conducted. This survey concluded with questions regarding motivation to participate and the experience of doing so. Of the 758 individuals who participated in the survey, 545 provided open-ended text responses to questions regarding motivation and 523 did so for questions regarding the experience of participating. It is these responses that are the focus of this paper. Data were analysed thematically. (3) Results: Motivations to participate were expressed as primarily altruistic in nature, with a future focus on improving the experience of the person who had attempted suicide alongside carers to ease distress. The experience of participating was difficult yet manageable, for all but a few participants. (4) Conclusions: With the increasing interest in first-hand accounts of suicide, how individuals experience participation in research is an important focus that requires further attention.

## 1. Introduction

Suicide is a significant public health challenge that can have lasting psychological, physical, and social consequences for those who are exposed to it [1]. Suicide is the 13th leading cause of death in Australia, resulting in over 3000 deaths in 2018 [2]. For every suicide there are approximately 25 suicide attempts [3]. This makes exposure to suicidal behaviour a relatively common occurrence, with approximately 58% of people in a representative Australian study indicating that they knew someone who had died by suicide [4]. This finding is supported elsewhere, and includes reporting across a wide range of relationships reflected within the proposed continuum of suicide exposure [5,6,7]. Less research has been published on exposure to suicide attempts. However, in a cross-sectional Australian community survey of exposure to suicide attempts and deaths [8], exposure to both was common (79.4%, compared with 13.7% reporting exposure to attempt only, and 6.9% reporting death exposure only). Previous research on suicide exposure has primarily focused on treatment-seeking kin, often identified and recruited via support groups or other therapeutic resources. This results in samples that are skewed towards participants interested in and actively accessing support [9,10]. Widely publicised online survey research allows for participation from broader sections of the community of interest, including non-treatment seeking individuals, people across the continuum of suicide exposure, and those who generally experience little distress or even resilience following the death [9]. 

Online surveys are likely to attract people who have an interest in this topic, yet present new challenges beyond those often reported in relation to other suicide research methodologies, which have resulted in delays to research projects or major changes to methodology to obtain ethical approval [11,12]. Nevertheless, in a study comparing online and offline survey research in general bereavement, the authors found online research to be valid and reliable [13]. The authors cite a number of benefits of online survey research, including convenience, reduced costs, increased access to a larger population, and increased access to a diverse range of people experiencing bereavement [13]. Online survey research also offers anonymity [13], which may encourage participation from stigmatised groups, including people exposed to suicide attempts and deaths. The anonymity of online research is seen as beneficial and may allow engagement from people who would otherwise not participate in face-to-face research [14]. Further, online research provides participants with a greater sense of control over sharing their experience [14], with an opportunity to pause their engagement and come back to a study at a later time if needed [15]. However, the online environment can result in lack of control of the study environment and no opportunity to monitor participants [13] or respond to distress.

Where experiences of people participating in trauma-focused research, such as suicide-related research, both online and in person, are reported, the research suggests that such participation is generally positive. Most participants report benefits from research participation, despite some experiencing distress [16,17,18,19,20], with benefits often outweighing any perceived costs [21]. However, there is also a small minority of people who may experience negative effects, though the research challenges assumptions that distress is always inherently negative [22]. Participants generally do not cite sad or painful memories evoked by the research as bad, and most negative experiences are temporary in nature [1,22]. Additionally, most participants do not regret their participation, despite experiencing distress [20,23,24]. This suggests that while participants may experience distress during the research process, they still report research participation as positive, and even helpful [1,19]. Furthermore, participants have reported additional benefits of research participation, including gaining insights from working through grief elements previously unaddressed and sharing their story with others. The latter point can contribute to increased understanding, meaning-making, and meaning reconstruction after a loved one’s death [22,25,26,27]. 

Evidence on how participants experience these events and what motivates them to participate in suicide-related research remains scant in the literature, particularly in research aimed at understanding firsthand accounts of suicide from the perspective of a person caring for someone who has previously attempted suicide. This manuscript focuses on answering two questions: (1) What motivates people to participate in suicide-related research? (2) What are their experiences of participation in research focused on understanding their lived experience of providing care to someone who had previously attempted suicide? These two questions were specifically included in a broader survey due to discussions with the university ethics committee during the approval process about the safety of asking carers about their experience, and lack of guidance in the existing evidence base to help address this concern. This experience with ethical review is not uncommon with suicide bereavement research [12] and suicide related research more broadly [11]. Yet, often the literature is focused on allaying ethical review boards’ concerns about the safety of the research, rather than investigating the experience of participants and their motivations for being involved. Thus, this paper provides insights into, and enhances our understanding of, participants’ motivations and lived experiences of taking part in research into exposure to suicidal behaviour.

## 2. Materials and Methods

An online survey was conducted between 27 November 2017 and 5 February 2018. The target audience was individuals who self-identified as being carers for individuals who had previously attempted suicide to understand the impact of caring and interactions with health services. See Coker and colleagues [28] for further information. The survey was advertised in Australia through a national mental health charity and associated networks websites and social media accounts (Facebook, Twitter). The University of New England (Australia) Ethics Committee (number HE17-210) granted ethical approval for the survey. A participation information sheet was provided by following the survey link, which outlined the purpose of the study, inclusion criteria, and support services available to the participant. This also included information about the right to withdraw from the survey at any time. The survey was made up of 54 questions relating to experiences of providing care to someone who had previously attempted suicide. This manuscript specifically focuses on the final two questions of the survey, which asked participants for open-ended text responses (no text limit) to the following two questions: (1) Please tell us what motivated you to participate in this research. (2) Can you tell us about your experience and/or the feelings and emotions you had as you participated in this research and were asked questions about suicide?

The data from the responses to each question were extracted from the full survey data to create two separate data files, one for each question. Each file was analysed independently by two coders thematically grouping similar responses together and identifying common and uncommon experiences. Data exploring individuals’ motivations for study participation were inductively blind coded by M.M. and A.S. to group like motivations together into themes. Where more than one theme applied to a statement, these were both coded. A third author (S.W.) then reviewed the themes, highlighting any discrepancies between the coders. These were then discussed among the three coders until consensus was achieved and themes agreed upon, with an overarching statement describing the theme developed.

Data from the question related to experiences of participating followed the same process where the data were inductively blind coded by two authors (M.M. and R.S.) to determine how the participant explained their experience of participating. These two authors then discussed the emerging themes to reach consensuses on themes and overarching statements to describe each theme. Finally, the themes derived from Questions 1 and 2 were reviewed by two authors (M.M., S.W.) to determine over overlapping or common threads between the responses on motivations for and experiences of the research participation. 

## 3. Results

The full sample comprised 834 participants who responded to the survey. A number of participants were excluded as the participants: did not provide consent (n = 5); were under 18 years of age (n = 14); resided outside Australia (n = 15); or either identified that they did not know a person who had attempted suicide or did not provide a response to this question (n = 42). Following these exclusions, the final sample comprised 758 participants. Of those 758 completing the survey, 547 responded to these open-ended questions; 545 provided a text response to the question asking about motivation to participate and 523 responded to the question on the experience of participating. As access to the survey was through an open link sent out via a number of agencies, we checked to determine whether any person participated more than once by checking postcode, year of birth, and gender. No response had the same information for each of these data points; thus, all responses were deemed unique. Demographic information about the participants is included in Table 1. 

In relation to the first question on participants’ motivations to participate, three themes were identified. These themes are altruistic in nature: (1A) Good data can be a powerful tool for change, (1B) participation as an attempt to ensure inclusion of lived experience, and (1C) motivation to effect change in the mental health system broadly, or to shift workforce views on responding to attempts. In relation to Question 2: Experiences of participating, four themes were identified. These are: (2A) importance of involvement, despite the fact that it elicited difficult emotions; (2B) involvement triggering painful memories and experiences; and (2C) safe reflecting on the process of providing care post suicide attempt, via survey participation. In addition, a final theme entitled (2D) outliers—concerning responses (without context)—also included concepts of note for further research. 

These questions and themes are presented in Table 2 with quotes from participants used to illustrate the thematic development throughout the results. These are presented verbatim from the text the participant entered into the survey without editing, with the exception of stigmatising language which has been changed in three instances to remove the word “commit” and to correct grammar or clarify meaning (indicated by square brackets in participants quotes). Participants are identified by gender and age and relationship to the person who attempted or died by suicide, to maintain anonymity but provide context to the quote. 

### 3.1. Question 1: Motivations to Participate

Question 1 relates to the data collected inquiring about the reasons participants gave for being motivated to participate upon seeing information about the study. All responses are grounded within an altruistic motivation to reduce the suffering of others. The first focused on being motivated to change public perceptions about suicide and the role of carers; the second to shed light on the reality of caring; and the third focused on being motivated to ensure others did not suffer through systemic issues as the participant had. Each theme is presented below. 

#### 3.1.1. Theme 1A. Good Data Can Be a Powerful Tool for Change

The first theme related to the participant’s motivation to take part in the research as a deliberate action to broadly contribute to suicide prevention and public health. This included participating to address the causes and consequences of stigma, and to assist with knowledge generation to enhance the development of suicide prevention strategies. Participants viewed reducing stigma as a mechanism for preventing suicide, which could facilitate more authentic dialogue around the causes and risk factors for suicidal behaviour.

“It is important to educate the community that suicide is real, and everyone is affected by it,”.

(female, 57 years, parent)

Commonly, participants wanted a change in how mental health and suicidal behaviour was viewed and treated by society and the health system. In the absence of speaking more openly about suicidal behaviour, some participants felt the problem would continue to worsen without any shift in incidence or impact.

“The more [suicidal behaviour] is shunned the more it will happen,”.

(female, 50 years, family relation)

Participants also articulated a sense of valuing their own health, and the health of others known—and unknown—to them. They wanted to ensure that those vulnerable to suicide bereavement or suicidal behaviour are protected from traumatic experiences that contribute to suicide.

“Anything that can help those struggling with living in this world is a good thing,”.

(female, 58 years, parent)

Motivations regarding the enhancement of knowledge of suicide to inform prevention efforts was reinforced.

“I worry for my friend. Suicide is the act of someone who doesn’t know what else to do, who feels they have no other option. There is always a better option. I want to help anyone who is struggling, to find a better option”.

(male, 42 years, friend)

In addition to impacting the lives of those in the future who may have lived experience of suicide, participants also reflected on the action of including individuals with lived experience of suicide in research: “Good data can be a powerful tool for change,” one participant, male, 62 years, family relation, explained. 

Given the prevalence of suicide in the community, participants expressed dismay at the lack of services and formal support available, viewing research participation as a way they could actively participate in challenging this situation.

“Too many people still can’t talk about [suicide] openly with the right people, either because the right people aren’t available or they’re too scared to talk to anyone. Research that helps to improve services and provide education to people is going to be helpful,”;

(female, 36 years, child)

and

“Research is vital to gain insight into mental health conditions, lived experience and service provision,”.

(female, 56 years, parent)

Overall, this theme highlighted that participants were motivated to participate in the research for suicide prevention purposes.

#### 3.1.2. Theme 1B: Motivated to Participate to Highlight the Lived Experience of Caring

This theme encompasses participants wanting to share their experiences of caring for someone who had attempted suicide. This was presented as (i) the intention of helping others experiencing poor mental health, and (ii) sharing with intent to depict carers’ experiences of day-to-day life with their respective loved ones who are/were engaging in suicidal behaviour. This was presented from the stance of having experienced their own respective difficulties and having been carers for people who had attempted suicide.

“I have a passion for helping, particularly with areas of mental health and suicide prevention as it is part of my ‘former life’. I no longer have suicidal thoughts but now I help others. Anything that helps, I will do,”.

(female, 49 years, parent)

Highlighting the experiences of carers, particularly those for whom the person had died by suicide, was particularly important in valuing the broad contribution their experience can have more broadly, as this parent described.

“Struggling daily since my son passed away and don’t want others in lived experience to have to go through this,”.

(female, 50 years, parent)

Those still providing care to an actively suicidal person spoke of the motivation to share their experience as a way to assist in coping with “*living with the daily fear*” that suicide attempts bring, and the resultant preoccupation about not if, but when, their respective family members would take their own lives.

“My son had his 4th attempt on the [date]. That made 4 attempts within the year. He states he will eventually [die by suicide]. It kills me knowing he will do it, but where and when […]”.

(female, 71 years, parent)

Yet others for whom the situation leading to suicidal crises had resolved wanted to share their hopefulness in the future following this period of caring.

“The person who attempted suicide is now my wife and we together we have faced things together. And we feel no shame or embarrassment over this event it did make [us] stronger and able to tell our story in which it has shown others there is light out of that black hole”.

(male, 41 years, friend)

Participants described that study participation had the potential to help with meaning-making around the attempted suicide or suicide death of their loved one. Particularly, participants longed to know why their loved one engaged in fatal or non-fatal suicidal behaviour, particularly to help “those they leave behind”—the family and friends who had cared for this person during their crisis; and to be able to shed light on the intimate struggles and challenges they had faced in providing care.

“Still need to understand how my husband got to the point of planning and executing his suicide [death]. None of the family had any idea. He has not opened up about it to any of his loving immediate family,”.

(female 64 years, partner)

#### 3.1.3. Theme 1C: Motivated to Participate by the Desire to See Change in Systems

Many participants were highly critical of the services and supports that were available to their family member to address suicidal crisis and caring needs. Desire for immediate action to change the health system to reduce suicide death rates and suicidal distress was frequently expressed. Participants wanted greater awareness among healthcare staff.

“My daughter has Anorexia Nervosa which limits our access to primary mental health services, is misunderstood by medical staff and in desperate need of research and funding in Australia. I am a lioness mother advocating for my daughter’s life!”.

(female, 56 years, parent)

On an individual level, participants reflected on the lack of formal supports for their loved one and felt they were supporting their family member alone. This was a clear motivation to take part in the research.

“I have support[ed] my son when no one else would; I had to educate myself to help support my son when we were turned away. Others should not have to go through this and help should not be determined on the $ [ability to pay for services],”.

(female, 49 years, parent)

Participants hoped that sharing this experience would save others experiencing something similar in future.

“We had to go out into the night to find him and get him to hospital in order to save his life. I hope doing this survey will mean that another family doesn’t have to do what we did,”.

(female, 59 years, parent)

Overall, participants’ reasons for participating in the research were underscored by altruistic motivations in a desire to reduce suicide-related distress in the community. This was expressed through increasing the research evidence base and understanding what it means to be a carer and how these experiences can better inform practice.

### 3.2. Question 2: Experience of Participating

The second question asked participants about their experience of participating in the survey. This re-focused the participants to the present, whereas the previous question asked about what motivated them to commence participation. While there is some overlap in the altruistic nature of participation, here the participants reflect on their emotions immediately post participation. The first three themes highlight the range of experience from the importance of participating in research even while difficult to do so through to the ways in which participating had triggered troublesome emotions. These three themes offer insight into the ability to reflect on both the challenges of recalling events related to caring for the person who had attempted suicide, and the nature of participation. The fourth theme presents data where there were statements, all negative in tone, but without additional contextual information to assist in coding. Nevertheless, these statements are included, as they provide additional insight into how some people do experience these kinds of surveys and offer an opposing perspective. Each theme is presented below.

#### 3.2.1. Theme 2A: Importance of Involvement, Despite the Fact that It Elicited Difficult Emotions

Participants frequently reported that while participating and recalling details of their caring journey may be difficult, this was superseded by the importance of participating for the betterment of care for people who will attempt suicide in the future and their carers. Participants reported a sense of hopefulness that their participation would lead to changes in the health system responding to individuals’ experiences and families exposed to suicidal behaviour. This altruistic motivation underlies the responses to Question 1, presented above; however, here this was tempered with the participant being asked to respond to questions about the experience of participating, thereby focusing on their current state at the end of the survey. 

Participants expressed gratitude that this research was happening and that they had an opportunity to contribute despite any pain it may cause.

“I felt a little emotional but it’s outweighed by knowing that this research can work to help better support others in similar positions. And a great weariness but also a sense of purpose in the need to share stories of survival and growth through encounters with mental disturbance. AND Hopeful that this will help others,”.

(female, 52 years, partner)

While recalling events was difficult, the emotional response was seen as a natural process when being asked specific questions about their experiences.

“[Experience of participating made me] Somewhat teary, which is still at times my reaction to talking about past issues. That feels natural. Teariness of short duration however,”.

(female 54 years, parent)

The gratitude felt because they were given an opportunity to participate, especially when linked to hoping that participation might positively influence those in the future.

“I am glad that I can have some input into helping professionals learn what works and what doesn’t work, when helping a suicidal person,”.

(female, 40 years, parent)

In particular, they reported wanting others to know about their experiences as carer and the difficulties they were encountering. In addition, being asked first-hand about their caring experiences led to gratitude.

“Pleased to see that finally such an organisation has realised that those closest to the at risk person need to be brought into the discourse around mental health,”.

(female, 65 years, parent)

For others, recalling their experiences in this way also helped them to think about the professionals working within the health system. This was particularly true for participants who were also working as health and social care professionals.

“It made me realise how little support there is for health professionals who are exposed to suicidal behaviour every day,”.

(female, 40 years, health provider)

Meanwhile, some participants offered a more sceptical view: that research such as this will never deliver tangible outcomes, yet they participated regardless, as this participant noted.

“Is this survey just another ’tick box’ or will something meaningful actually come out of it? I guess a sense of hopelessness. There is too much PR spin and not enough actual outcomes,”.

(female, 46 years, friend)

This theme provides some insight into participants’ experiences of participation somewhat mirroring their motivations (as seen in Question 1 data), those being primarily altruistic.

#### 3.2.2. Theme 2B: Safe Reflecting on the Process of Providing Care Post Suicide Attempt, via Survey Participation

The second theme offers insight into a more internally reflective state as an outcome of participation. Most often this was due to the nature of the caring role, and so participation resulted in the participant being reminded of the daily challenges in caring for someone who was, or is currently, suicidal, and how difficult this is for the suicidal person as well as those around them.

“Lump in my throat, felt compassion for my friend and others going through hard times,”.

(female, 38 years, friend)

Empathy for those still struggling everyday was expressed, as was concern for others who may be more vulnerable at the time of participation.

“I feel sad because there will be participants completing this questionnaire who are still raw in the emotions and may not have adequate support systems in place. I worry that it may trigger someone and they won’t take the questionnaires suggestions to call a support service for help,”.

(female, 44 years, parent)

Some participants recognised that the survey would likely have been more difficult if they completed it earlier when their caring role was still active at the time of acute crisis; that time had given them space to be more reflective.

“Returned to feelings I experienced at time of attempted suicide—confusion, dreamlike, inadequacy, overwhelmed, tiredness, sadness and grief. As time has passed I view this time and feelings almost as an objective observer,”.

(female, 63 years, parent)

This sentiment was reported by others who reported being able to cope with distressing emotions prompted by the survey questions because of the resources, tools, and skills they had developed through being carers, describing this as the ability to be.

“Detached, analytical, I am able to suppress the emotional aspect to answer the questions without letting them affect me”.

(male, 50 years, family relation)

Yet others recognised that emotional states can fluctuate at any given time. Importantly, this participant also noted the contact numbers provided throughout the survey should they need them.

“I felt very reflective with the whole process. I felt ‘safe’ [and] interestingly [today] has been a ‘good day’ for me, so I felt in a good calm space mentally, physically & emotionally all the way [through] it, whereas normally my pain levels [and] mood swings erratically daily…I appreciated how the various prompts at different stages gave the different phone numbers to call if I needed to talk to someone,”.

(female, 40 years, sibling)

For other participants, the survey was a reminder about people they care about who are currently struggling; one that often suicide risk remains current even when time passes.

“My daughters last attempt (to my knowledge) was 2 years ago. I am vigilant every day to changes in her mood & behaviour. I worry about her constantly. I have done a mental health first aid course to try and equip myself. I still feel vulnerable, I still cry when I think about it all. Even 2 years on when she is now managing her depression and anxiety better, I cannot relax. Our lives will never be the same,”.

(female, 52 years, parent)

Where the person had died, participation also caused a reflective state, but this was focused on the loss of the person and their future.

“Sad to reflect on the people whom I have lost to death by their own hand … sad for them. for whatever they may be experiencing now and for all that they have missed in the lives of their loved ones”.

(male 53 years, stepson)

Throughout the survey, reminders of 24/7 crisis line phone numbers were provided, as is standard practice. These were viewed as a useful tool to help support the participant through completion of the survey, even where they did not use them.

“Overall I was able to go from one question to the next or one section to the next. Your warnings/tips/support message at the beginning and throughout the survey were a big factor in this. There were moments, that I needed to breathe and stop before I continued. For some it was the distress of the incident, others it was validation of self … This survey gave me the opportunity to acknowledge myself and each person I have supported,”.

(female, 49 years, friend)

In addition to tapping into these difficult reflections, participants also expressed feelings of relief and validation for their experiences as a result of completing the survey, as well as solidifying their experience.

“I thought a lot about my past experiences and how hopeless and sad they’ve made me feel. But this survey also makes me feel a bit more validated in how drained I feel being a support person,”.

(female 23 years, friend)

For many this was expressed from a point of reflection on how far they had come.

“It touches my heart, it soothes the nerves, I can have a voice, and that gives me hope. I cried a little, I sighed some more, and sometimes had a smile to remember the times I have survived through to get to here,”.

(female, 56 years, parent)

A variety of emotional states were reflected upon by participants. These were closely linked to where they were at with the caring role and how they were able to reflect on this with some emotional distance, even while empathic to others. 

#### 3.2.3. Theme 2C: Involvement Triggering Painful Memories and Experiences

This theme highlights that research such as this does cause participants to draw on painful experiences that may trigger past difficult memories and emotions into the present. Some respondents reported that any reminder related to their experience was challenging. These responses differed from those who expressed reminders of being upsetting but could reflect on them as in the prior theme, as these responses were very specific feelings, reactions, and thoughts that were brought back into the present, without the participant extending their statement to include any reflective text, as demonstrated in this parent’s response.

“The survey has brought back the feelings of fear, loneliness, helplessness and hopelessness that I felt at the time of my son’s suicide attempts,”.

(female, 58 years, parent)

Text appeared to demonstrate that participating in the survey was a triggering event tapping into past traumatic emotional states.

“I felt very sad, and kind of hopeless. Almost like I was reliving the event again,”.

(female, 30 years, sibling)

For one individual, participation brought up a difficult situation she was still experiencing with thoughts of what might occur in the future.

“Brought it all back. Upsetting. Feel hopeless coz she won’t talk about it to me. So I’m just waiting for a call all the time. When she walks out the door “to get some air” I never know if she’s coming back. So I live in fear to some extent. Then try to get on with normal things but my gut and my brain both know I’m PRETENDING. All the Time. I’m not calm. I’m just passing time productively and hiding all my pills from her until that evil slime smothers my beautiful daughter again,”.

(female, 54 years, parent)

While there were fewer participants who expressed they were emotionally triggered by participation, it is important to highlight that for some participation in this type of research was difficult. 

#### 3.2.4. Theme 2D: Outliers—Concerning Responses (Without Context)

This final theme presents short statements from several participants (n = 15) which could not be coded into the previous themes. This is where participants responded briefly to the question about their experience of participating with responses, all referring to experiences of completing the survey but without context to be able to adequately code. While these may relate to Theme C, there was not enough text provided by the participant to be able to adequately code. Examples of some of these are provided below.

“A little scary as it brought back memories,”.

(female, 63 years, sibling)

“It brought up some suppressed emotions and at multiple points I considered closing the browser and not completing”.

(male, 33 years, sibling)

“Reminded me of a very tough time in my life—was a bit anxious and at times thought about discontinuing the survey,”.

(female 44 years, parent)

While only few in number, without this being a mandatory question to complete the survey, it is possible others felt similarly challenged by completing this survey but did not express this, or may have terminated their involvement prior to completion of the survey, which is where these final questions were located.

## 4. Discussion

The data were captured via two questions at the end of a survey for participants who self-identified as having provided care for individuals who had made at least one suicide attempt each. These two questions aimed to understand and provide insights into the motivations for, and experiences of, participating in this research. This came from concerns raised during the ethical approval process regarding participant safety. It is not uncommon to be faced with such concerns in suicide research [11,12], with researchers reporting that ethical review boards require modifications and restrictions to the proposed studies due to concerns that asking about suicide might exacerbate risk [9,12], yet this concern is primarily unfounded by previous research [29,30]. 

Literature reporting on the motivation for participating in suicide-related research suggests the dominance of altruism and a desire to help others. This is characterised by expressing hope that sharing their story will contribute to research that will help advance the field to better support others [22,26] or prevent suicide [27]. Altruism and a desire to ease others’ future difficulties consistently emerges as a motivation for research on topics beyond suicide [31,32,33]. For example, in a study examining the ethics of general bereavement research, the authors found that bereaved adults felt positive about bereavement research, reporting perceived benefits in line with altruistic motivations [34]. Treatment focused research also suggests that some people are motivated by the hope that research participation will benefit them in personal, direct ways, such as access to treatments [32], a phenomenon termed “conditional altruism” [35]. 

The findings presented here suggest further nuance. That is, while motivations were primarily altruistic, being for broader improvement for suicide research, policy, and practice, there was also a focus on how participating offered an opportunity to reflect on the experiences of the past. This suggests a meaning-making component, with the gain being internal growth, not material. 

In terms of experiences of research participation, the findings of this study echo previous research, suggesting that participation was generally deemed as positive, even if they experienced distress at the time [1,19]. Importantly, participants expressed validation and gratitude for the opportunity to contribute. This also presents an opportunity to share their stories that may not otherwise exist, an opportunity that is inhibited by “overprotective gate-keeping” [36] (p. 356). Participants reported greater insight and awareness resulting from their responses to the questions, supporting previous research, which has found a therapeutic benefit to research participation. Such research suggests that the therapeutic value of research participation extends to non-traumatically bereaved participants [37] and participants who have survived a suicide attempt or report self-harm [14,36].

Ethical review boards continue to make assumptions about the vulnerability of people exposed to suicide, reinforcing notions of risk that can contribute to stigmatisation [12]. They cite concerns about the potential of research to retraumatise participants, which demonstrates a fundamental misunderstanding of the nature of trauma [20,24], and runs in direct contradiction to the lived experience of many research participants. In one study, bereaved parents “felt that because they were thinking of their child every day, there was not added danger in talking about it” [25] (p. 398). Furthermore, within and outside of suicide-focused research, researchers have challenged notions that vulnerable populations have impaired decision-making capacity and are not able to freely choose to participate [38], evaluate their capacity for participation [39] or provide informed consent [14]. 

While many participants reported manageable reactions to participating, negative emotions were reported by a few participants, for whom partaking in the survey did trigger distressing emotions and thoughts. Whilst there were instances where the participants noted that the questions prompted revisiting painful periods, they went on to complete the survey. This suggests that for the vast majority, experiencing negative emotions was not perceived as a valid reason for partial or non-completion of the research. It is plausible that participants may have anticipated some degree of upset or discomfort prior to beginning the survey and viewed this as part of their lived experience. The inclusion of crisis support numbers was mentioned as a useful reminder that support was available. Nevertheless, a small number of participants did state that their experience was challenging, and without ways to contact them post survey, this does provide a salient reminder that participating in such research may be potentially harmful for some. Yet, it is difficult to identify those who may have this experience following survey participation, and that should remain a consideration for this type of research.

Future research should increase our understanding of who is negatively affected by online survey participation and whether screening mechanisms could be put in place to minimise the potential for harm. Instead of engaging in restrictive protocols or paternalistically determining who can and cannot participate in research, one suggestion is to develop questions that prompt reflection, insight, and self-awareness so that potential participants are able to determine if they are ready and willing to engage. Understanding the role and capacity of referral options, offered to individuals who take part in studies, needs attention. Post-survey completion tracking via electronic information from internet servers is unlikely to result in support in a timely manner and would lead to issues regarding anonymity. Alternatively, a distress protocol which asks participants to confirm periodically if they are still interested in continuing with logic set-up to redirect to the end of the survey if selected may offer distressed participants an exit strategy from the survey, along with timely information about support. Some examples of this are presented by Draucker, Martsolf, and Poole [40], who created two distress protocols for interviews with potentially vulnerable populations, one being a screening stage and the other for responding to distress during the interview. While the protocols were created for studies where the researcher has direct contact with participants, future research could assess whether tools such as this might be able to be adapted to online surveys. 

As with all research, these findings are limited by the design of the survey. This paper focuses on just two questions aimed at exploring the experience of participation in an online survey for people who have cared for someone who had previously attempted suicide. This was a cross-sectional community survey, so is unlikely to be representative of all experiences. Importantly, our sample is heavily skewed toward female participation, as has been reported across suicide exposure research previously [9]. In that review, the range of female participation was between 60 and 90 percent, so our sample here is at the upper end of that range. Nevertheless, these data do provide some insight into key considerations for understanding why people choose to participate in survey research and their experiences of doing so. 

We have an ethical imperative to engage in research with vulnerable populations to advance treatment options and understanding [20,38]. Any attempt at gatekeeping reflects a violation of ethical principles related to autonomy and justice [41], as it prevents individuals from experiencing possible research benefits [36]. While ethical review boards have a responsibility to ensure protection of research participants from harm, it is imperative that ethical responsibilities are balanced with our knowledge of benefits derived from participation and respect for the autonomy of participants to make a choice for themselves [24]. Recognising the inherent ethical challenges of any research approach, it is suggested that rather than restricting methodology, methods, and processes for support, engagement should be encouraged [39] to ensure protection for vulnerable populations. 

## 5. Conclusions

This study furthers our understanding of the experience of participation in survey research, through the inclusion of people exposed to suicide. The study reinforces that underpinning all ethical suicide research is the premise of “do no harm,” and that any risk to a person’s wellbeing is limited. However, there is a need to recognize that these participants would likely have experienced trauma as a result of their lived experience of suicide. This study presents a novel understanding of the motivations for, and experiences of, participating in an online survey related to suicide exposure. It identifies that online survey research comes with many benefits, as it enables participation from a wide range of individuals impacted by suicide, in addition to treatment-seeking kin. This expands our understanding of suicide exposure by including the perspectives of people providing care to individuals who have attempted suicide, a population often neglected in the literature.

## Figures and Tables

**Table 1:** Demographic information of participants (N = 547 *).

Characteristic	N (%)
Geographic Region	
Metropolitan	282 (51.6%)
Regional	174 (31.7%)
Rural	84 (15.4%)
Remote	7 (1.3%)
Gender	
Female	479 (87.7%)
Male	59 (10.6%)
Other	9 (1.7%)
Age	
18–24	24 (4.4%)
25–34	63 (11.6%)
35–44	100 (18.3%)
45–54	187 (33.9%)
55–64	132 (24.2%)
65+	41 (7.5%)
Relationship to Person Who Attempted Suicide	
Child	167 (30.6%)
Friend	115 (21.1%)
Partner	69 (12.7%)
Parent	64 (11.7%)
Sibling	37 (6.8%)
Family relation (cousin, aunt, etc.)	37 (6.8%)
Colleague	7 (1.3%)
Other	51 (9.0%)

* 16 participants provided only their motivation and two participants provided only their experience; the remainder completed both questions.

**Table 2:** Overview of themes.

Survey	Survey Questions Used in This Analysis	Themes Identified	Brief Description of Each Theme
Online suicide research participation	Question 1: Motivation to participate	Theme 1A: Good data can be a powerful tool for change	Participating to break down stigma, use their experience to inform research
		Theme 1B: Highlight lived experience of caring	Participating to shed light on reality of lived experience
		Theme 1C: The desire to see change in systems	Participating to change systems and reduce suffering
	Question 2: Experience of participating	Theme 2A: Important, even though it is difficult	Experience was difficult but it was too important so the difficulty was worth it
		Theme 2B: An opportunity for reflection	Experience was reflective on personal progress and growth
		Theme 2C: Reminders and triggers	Experience did remind me of pain and was triggering of difficult emotions
		Theme 2D: Outliers	Concerning responses to participation without additional explanation

## References

[1] Andriessen K., Krysinska K., Draper B., Dudley M., Mitchell P.B. (2018). Harmful or Helpful? A systematic review of how those bereaved through suicide experience research participation. Crisis.

[2] Australian Bureau of Statistics Main Features—Australia’s Leading Causes of Death, 2018. https://www.abs.gov.au/ausstats/abs@.nsf/0/47E19CA15036B04BCA2577570014668B?Opendocument.

[3] Drapeau C., McIntosh J. USA Suicide: 2017 Official Final Data. https://www.suicidology.org/Portals/14/docs/Resources/FactSheets/2017/2017datapgsv1-FINAL.pdf.

[4] Maple M., Sanford R., Pirkis J., Reavley N., Nicholas A. (2019). Exposure to suicide in Australia: A representative random digit dial study. J. Affect. Disord..

[5] Cerel J., Maple M., van de Venne J., Moore M., Flaherty C., Brown M. (2016). Exposure to suicide in the community: Prevalence and correlates in one US state. Public Health Rep..

[6] Feigelman W., Cerel J., McIntosh J., Brent D., Gutin N. (2017). Suicide exposures and bereavement among American adults: Evidence from the 2016 General Social Survey. J. Affect. Disord..

[7] Cerel J., McIntosh J., Neimeyer R., Maple M., Marshall D. (2014). The continuum of “survivorship”: Definitional issues in the aftermath of suicide. Suicide Life-Threat. Behav..

[8] Maple M., Sanford R. (2019). Suicide exposure and impact within a non-representative Australian community sample. Death Stud..

[9] Maple M., Cerel J., Jordan J.R., McKay K. (2014). Uncovering and identifying the missing voices in suicide bereavement. Suicidol. Online.

[10] Maple M., Pearce T., Sanford R., Cerel J., Castelli Dransart D.A., Andriessen K. (2017). A systematic mapping of suicide bereavement and postvention research and a proposed strategic research agenda. Crisis.

[11] Andriessen K., Reifels L., Krysinska K., Robinson J., Dempster G., Pirkis J. (2019). Ethical concerns in suicide research: Results of an international researcher survey. J. Empir. Res. Hum. Res. Eth..

[12] Moore M., Maple M., Mitchell A.M., Cerel J. (2013). Challenges and opportunities for suicide bereavement research: The experience of ethical board review. Crisis.

[13] Tolstikova K., Chartier B. (2010). Internet method in bereavement research: Comparison of online and offline Surveys. OMEGA J. Death Dying.

[14] Gibson S., Boden Z., Benson O., Brand S. (2014). The Impact of Participating in Suicide Research Online. Suicide Life-Threat. Behav..

[15] Lefever S., Dal M., Matthíasdóttir Á. (2007). Online data collection in academic research: Advantages and limitations. Br. J. Educ. Technol..

[16] Ferrier-Auerbach A.G., Erbes C.R., Polusny M.A. (2009). Does trauma survey research cause more distress than other types of survey research?. J. Trauma. Stress.

[17] Goossens I., Nicholls T., Torchalla I., Brink J., de Ruiter C. (2016). The perceived impact of trauma-focused research on forensic psychiatric patients with lifetime victimization histories. J. Empir. Res. Hum. Res. Eth..

[18] Griffin M., Resick P., Waldrop A., Mechanic M. (2003). Participation in trauma research: Is there evidence of harm?. J. Trauma. Stress.

[19] Jorm A.F., Kelly C.M., Morgan A.J. (2007). Participant distress in psychiatric research: A systematic review. Psychol. Med..

[20] Legerski J.-P., Bunnell S.L. (2010). The risks, benefits, and ethics of trauma-focused research participation. Eth. Behav..

[21] Ruzek J., Zatzick F. (2000). Ethical considerations in research participation among acutely injured trauma survivors: An empirical investigation. Gen. Hosp. Psychiatry.

[22] Omerov P., Steineck G., Dyregrov K., Runeson B., Nyberg U. (2014). The ethics of doing nothing. Suicide-bereavement and research: Ethical and methodological considerations. Psychol. Med..

[23] Kassam-Adams N., Newman E. (2005). Child and parent reactions to participation in clinical research. Gen. Hosp. Psychiatry.

[24] Newman E., Kaloupek D.G. (2004). The risks and benefits of participating in trauma-focused research studies. J. Trauma. Stress.

[25] Dyregrov K. (2004). Bereaved parents’ experience of research participation. Soc. Sci. Med..

[26] Dyregrov K., Dieserud G., Straiton M., Rasmussen M., Hjelmeland H., Knizek B., Leenaars A. (2011). Motivation for research participation among people bereaved by suicide. OMEGA J. Death Dying.

[27] Wong P., Chan W., Beh P., Yau F., Yip P., Hawton K. (2010). Research participation experiences of informants of suicide and control cases: Taken from a case-control psychological autopsy study of people who died by suicide. Crisis.

[28] Coker S., Wayland S., Maple M., Blanchard M. (2019). Better support: Understanding the needs of family and friends when a loved one attempts suicide. Techn. Rep..

[29] Dazzi T., Gribble R., Wessely S., Fear N.T. (2014). Does asking about suicide and related behaviours induce suicidal ideation? What is the evidence?. Psychol. Med..

[30] Mathias C., Michael Furr R., Sheftall A., Hill-Kapturczak N., Crum P., Dougherty D. (2012). What’s the harm in asking about suicidal ideation?. Suicide Life-Threat. Behav..

[31] Campbell H., Raisch D., Sather M., Warren S., Segal A.R. (2007). A comparison of veteran and nonveteran motivations and reasons for participating in clinical trials. Mil. Med..

[32] Chong S.-A., Ong Y., Subramaniam M., Abdin E., Marx C., Campbell A. (2009). An assessment of the understanding and motivations of patients with schizophrenia about participating in a clinical trial. Contemp. Clin. Trials.

[33] Haack L., Gerdes A., Lawton K. (2014). Conducting research with latino families: Examination of strategies to improve recruitment, retention, and satisfaction with an at-risk and underserved population. J. Child Fam. Stud..

[34] Beck A., Konnert C. (2007). Ethical issues in the study of bereavement: The opinions of bereaved adults. Death Stud..

[35] Midgley N., Isaacs D., Weitkamp K., Target M. (2016). The experience of adolescents participating in a randomised clinical trial in the field of mental health: A qualitative study. Trials.

[36] Biddle L., Cooper J., Owen-Smith A., Klineberg E., Bennewith O., Hawton K., Kapur N., Donovan J., Gunnell D. (2013). Qualitative interviewing with vulnerable populations: Individuals’ experiences of participating in suicide and self-harm based research. J. Affect. Disord..

[37] Germain A., Mayland C., Jack B. (2016). The potential therapeutic value for bereaved relatives participating in research: An exploratory study. Palliat. Support. Care.

[38] Nugent A., Miller F., Henter I., Zarate A. (2017). The ethics of clinical trials research in severe mood disorders. Bioethics.

[39] Walker E., Newman E., Koss M., Bernstein D. (1997). Does the study of victimization revictimize the victims?. Psychiatry Prim. Care.

[40] Draucker C., Martsolf D., Poole C. (2009). Developing distress protocols for research on sensitive topics. Arch. Psychiatr. Nurs..

[41] Lakeman R., McAndrew S., MacGabhann L., Warne T. (2013). ‘That was helpful … no one has talked to me about that before’: Research participation as a therapeutic activity. Int. J. Mental Health Nurs..

